# Synthesis of AgN_5_ and its extended 3D energetic framework

**DOI:** 10.1038/s41467-018-03678-y

**Published:** 2018-03-28

**Authors:** Chengguo Sun, Chong Zhang, Chao Jiang, Chen Yang, Yang Du, Yue Zhao, Bingcheng Hu, Zhansheng Zheng, Karl O. Christe

**Affiliations:** 1 School of Chemical Engineering, Nanjing University of Science and Technology, Nanjing, Jiangsu 210094 China; 2 School of Chemical Engineering, University of Science and Technology Liaoning, Anshan, Liaoning 114051 China; 3 Department of Chemistry, University of Southern California, Los Angeles, CA 90089-1661 USA

## Abstract

The pentazolate anion, as a polynitrogen species, holds great promise as a high-energy density material for explosive or propulsion applications. Designing pentazole complexes that contain minimal non-energetic components is desirable in order to increase the material’s energy density. Here, we report a solvent-free pentazolate complex, AgN_5_, and a 3D energetic-framework, [Ag(NH_3_)_2_]^+^[Ag_3_(N_5_)_4_]ˉ, constructed from silver and cyclo-N_5_ˉ. The complexes are stable up to 90 °C and only Ag and N_2_ are observed as the final decomposition products. Efforts to isolate pure AgN_5_ were unsuccessful due to partial photolytical and/or thermal-decomposition to AgN_3_. Convincing evidence for the formation of AgN_5_ as the original reaction product is presented. The isolation of a *cyclo*-N_5_ˉ complex, devoid of stabilizing molecules and ions, such as H_2_O, H_3_O^+^, and NH_4_^+^, constitutes a major advance in pentazole chemistry.

## Abstract (web summary)

The pentazolate anion shows great promise for high energy density materials, but has only been isolated alongside non-energetic counter ions that impede its energetic properties. Here, the authors synthesize a silver *cyclo*-N_5_-complex devoid of stabilizing ions, as well as a complementary [Ag(NH_3_)_2_]^+^[Ag_3_(N_5_)_4_]^−^ 3D framework.

## Introduction

The pentazolate anion, *cyclo*-N_5_ˉ, has recently been stabilized as (N_5_)_6_(H_3_O)_3_(NH_4_)_4_Cl^1^ and Co(N_5_)_2_(H_2_O)_4_·4H_2_O^2^. This discovery has received much attention due to the potential applications of *cyclo*-N_5_ˉ in high-energy density materials (HEDMs) and as a starting material for the syntheses of inorganic ferrocene analogs. However, these *cyclo*-N_5_ˉ complexes contained non-energetic counter ions or groups to enhance their stability, thus impacting their energetic properties. The successful synthesis of an essentially naked cyclo-N_5_ˉ salt still has a huge challenge for the fascinating pentazole chemistry and related materials science.

HEDMs require both low sensitivity and high performance^3^. Polynitrogen compounds hold great promise due to their fast energy release and eco-friendly decomposition products^4–7^. Major advances in this area have been made during the past two decades, the two most remarkable new species discovered in this field are the pentazenium cation, N_5_^+^,^6–8^ and the pentazolate anion, *cyclo*-N_5_ˉ^9–12^. However, the reported N_5_^+^ and *cyclo*-N_5_ˉ complexes generally contain non-energetic counter ions or groups to enhance their stabilities. For example, SbF_6_ˉ or SnF_6_^2^ˉ are non-energetic counter ions in N_5_^+^ salts^13,14^, and H_2_O, Clˉ, NH_4_^+^, and H_3_O^+^ are used to stabilize the *cyclo*-N_5_ˉ anion^1^. These non-energetic components impact their energetic properties, such as heat of formation and detonation parameters. Therefore, it is important to reduce or eliminate these non-energetic components.

As part of our long-continued research, here, we report the synthesis of a water-stabilized *cyclo-*N_5_ˉ salt, [Mg(H_2_O)_6_]^2+^[(N_5_)_2_(H_2_O)_4_]^2^ˉ, in which the non-energetic Clˉ of (N_5_)_6_(H_3_O)_3_(NH_4_)_4_Cl was removed. For the elimination of the water, a silver *cyclo*-N_5_**ˉ** complex (AgN_5_) was precipitated by the addition of AgNO_3_ to the [Mg(H_2_O)_6_]^2+^[(N_5_)_2_(H_2_O)_4_]^2^ˉ solution. By treatment with NH_3_·H_2_O, this AgN_5_ complex was converted to a 3D-framework [Ag(NH_3_)_2_]^+^[Ag_3_(N_5_)_4_]**ˉ** salt, which was characterized by its crystal structure. The AgN_5_ complex is stable up to 90 °C, is photolytically unstable decomposing to AgN_3_ and N_2_, and Ag and N_2_ are its only final decomposition products. The isolation of a silver *cyclo*-N_5_**ˉ** complex, devoid of stabilizing molecules and ions, such as H_2_O, H_3_O^+^, and NH_4_^+^, constitutes a major advance in pentazole chemistry.

## Results

### Materials synthesis and structural design

**Fig. 1:**
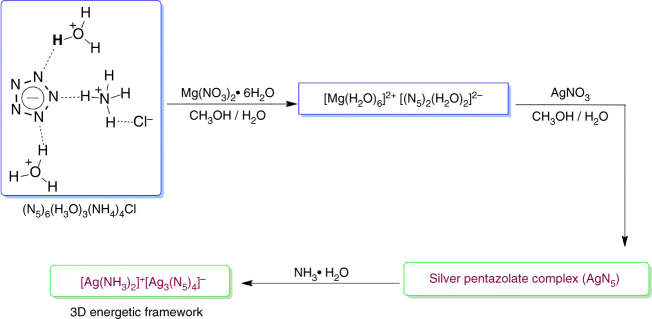
Syntheses of the silver pentazolate (AgN_5_) complex and [Ag(NH_3_)_2_]^+^[Ag_3_(N_5_)_4_]ˉ. Synthesis of AgN_5_ contains two steps by salt metathesis: The First-step of removing the non-energetic Clˉ and the second step of eliminating the H_2_O

**Fig. 2:**
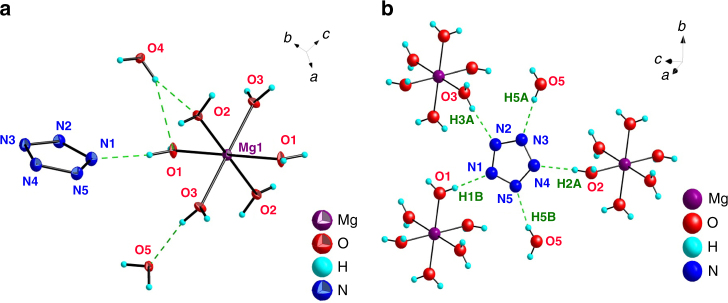
Crystal Structure of [Mg(H_2_O)_6_]^2+^[(N_5_)_2_(H_2_O)_4_]^2ˉ^. **a** ORTEP plot at the 50% probability level. **b** Coordination geometry of the *cyclo*-N_5_ˉ anion

The schematic in Fig. 1 illustrates the procedures for the syntheses of the AgN_5_ and [Ag(NH_3_)_2_]^+^[Ag_3_(N_5_)_4_]ˉ. In view of previous research, our team have achieved a breakthrough in *cyclo*-N_5_ˉ chemistry involving the synthesis and characterization of the stable pentazolate salt, (N_5_)_6_(H_3_O)_3_(NH_4_)_4_Cl^1^. We also demonstrated that a cobalt ion can effectively trap *cyclo*-N_5_ˉ, forming the stable compound Co(N_5_)_2_(H_2_O)_4_·4H_2_O^2^. As part of our continuing effort to prepare an essentially naked *cyclo*-N_5_ˉ salt, we first added magnesium nitrate to an aqueous solution of (N_5_)_6_(H_3_O)_3_(NH_4_)_4_Cl at room temperature, resulting in the formation of a white crystalline precipitate of [Mg(H_2_O)_6_]^2+^[(N_5_)_2_(H_2_O)_4_]^2^ˉ (Fig. 2) in 85% yield based on the *cyclo-*N_5_ˉ content of (N_5_)_6_(H_3_O)_3_(NH_4_)_4_Cl. Subsequently, an aqueous solution of silver nitrate was added dropwise to the stirred [Mg(H_2_O)_6_]^2+^[(N_5_)_2_(H_2_O)_4_]^2^ˉ solution in methanol, resulting in the precipitation of the AgN_5_ complex as a pale white solid. However, the AgN_5_ complex was light-sensitive and insoluble in all solvents tested. To further characterize this complex, we instantly treated it with 10 equiv. of NH_3_·H_2_O (25 wt%) at 0 °C, followed by warming to room temperature to liberate NH_3_ and to provide colorless crystals of [Ag(NH_3_)_2_]^+^[Ag_3_(N_5_)_4_]ˉ (Supplementary Fig. 1). This compound is thermally stable up to about 90 °C, where it starts to decompose with N_2_ evolution to form AgN_3_. In contrast to the very sensitive AgN_5_/AgN_3_, it is only moderately sensitive to impact and friction, with *H*_50_ = 73.8 cm (average) and an explosive probability *P* (%) = 76 (Supplementary Table 1), respectively.

### Crystal structure

The intermediate synthesis of [Mg(H_2_O)_6_]^2+^[(N_5_)_2_(H_2_O)_4_]^2^ˉ is an effective step to get rid of the Clˉ present in the original (N_5_)_6_(H_3_O)_3_(NH_4_)_4_Cl salt. The crystal structure of the above Mg salt was determined by single-crystal X-ray diffraction (Fig. 2a, b), which showed that it crystallizes in the triclinic space group *P*-1. The magnesium center is coordinated to six water molecules in an octahedral fashion with no direct bonding interaction between Mg^2+^ and *cyclo*-N_5_ˉ, in contrast to the structure of Co(N_5_)_2_(H_2_O)_4_·4H_2_O, where the cobalt ion acts as a shared center linking two pentagonal N_5_ˉ rings through two σ-bonds. Interestingly, the *cyclo*-N_5_ˉ ring is surrounded by five crystallographically independent H_2_O molecules, forming the water-stabilized *cyclo*-N_5_**ˉ** salt. Each of these bridging water molecules acts as an H-bond donor for a nitrogen atom of *cyclo*-N_5_**ˉ**. Such a coordination mode for *cyclo*-N_5_ˉ is unique and is of vital importance for the further construction of novel pentazolate complexes, because the stability of the water-stabilized *cyclo*-N_5_ˉ salt is determined primarily by hydrogen bonding. These hydrogen bonds can be relatively easily broken, if other cations can trap the *cyclo*-N_5_ˉ anion by forming strong chemical bonds.

**Fig. 3:**
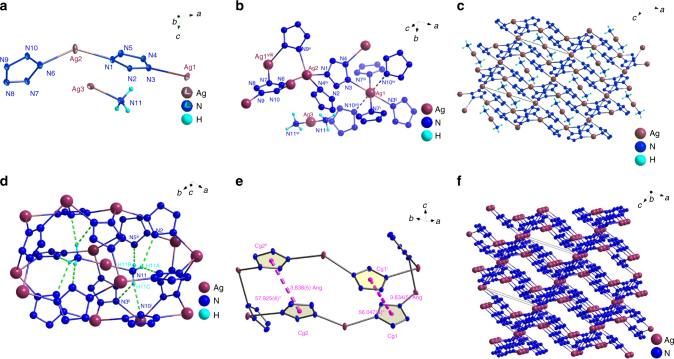
Crystal structure of [Ag(NH_3_)_2_]^+^[Ag_3_(N_5_)_4_]ˉ. **a** ORTEP plot of [Ag(NH_3_)_2_]^+^[Ag_3_(N_5_)_4_]ˉ at the 50% probability level. **b** The coordinate diagram of [Ag(NH_3_)_2_]^+^[Ag_3_(N_5_)_4_]ˉ. **c** Unit cell view along the *b* axis. **d** Schematic representation of the hydrogen-bonded motifs in the crystal structure: H-bonds are indicated as dotted lines. **e** π–π stacking interaction in the crystal structure (Cg1 and Cg2 were the centers of cyclo-N_5_ˉ). **f** The 3D framework of [Ag(NH_3_)_2_]^+^[Ag_3_(N_5_)_4_]ˉ

These considerations have sparked our interest in the preparation of other novel *cyclo*-N_5_ˉ complexes. As a consequence, we synthesized the AgN_5_ complex and its 3D-framework complex, [Ag(NH_3_)_2_]^+^[Ag_3_(N_5_)_4_]ˉ. The structure of [Ag(NH_3_)_2_]^+^[Ag_3_(N_5_)_4_]ˉ was determined by single-crystal X-ray diffraction analysis. It crystallizes in the monoclinic space group *P*2_1_*/c* with a calculated density of 3.2 g/cm^3^ at 123 K (Supplementary Tables 2–6). The density value is the highest crystal density reported so far for any *cyclo*-N_5_ˉ complex^15^, and is largely due to the presence of four heavy silver atoms. As depicted in the Oak Ridge Thermal Ellipsoid plot (ORTEP) of [Ag(NH_3_)_2_]^+^[Ag_3_(N_5_)_4_]ˉ (Fig. 3a), the asymmetrical unit contains half of an [Ag(NH_3_)_2_]^+^[Ag_3_(N_5_)_4_]ˉ molecule, which is composed of two Ag(I) cations (50% occupancy for Ag1 and Ag3, 100% occupancy for Ag2), two cyclo-N_5_ˉ rings, and one coordinated NH_3_ molecule. One *cyclo*-N_5_ˉ ring is no longer perfectly planar, showing a small degree of distortion, as evident from the torsion angles of N(6)-N(7)-N(8)-N(9) being –0.3° and N(8)-N(9)-N(10)-N(6) being 0.2°. In contrast, the other *cyclo*-N_5_ˉ ring resists distortion from planarity, causing a change in the N–N bond lengths (1.323–1.336 Å), which are slightly longer than the N–N bonds (1.318–1.320 Å) in Co(N_5_)_2_(H_2_O)_4_•4H_2_O. Figure 3b shows the coordination environment of the Ag cations. There are three crystallographically independent Ag centers in the structure. Ag3 is bridging between two ammonia molecules in a linear configuration with relatively short Ag3–N distances (2.110 Å; N(11)-Ag(3)-N(11), 180°). Ag2 is coordinated by four *cyclo*-N_5_ˉ rings, where four N atoms (N1, N4, N6, N9) adopt a distorted tetrahedral configuration around Ag2, with intermediate Ag2–N distances ranging from 2.332 to 2.370 Å, whereas Ag1 is surrounded by three pairs of *cyclo*-N_5_ˉ rings (N3, N7, and N10) adopting an octahedral geometry with two *cyclo*-N_5_ˉ rings at the apical positions and four *cyclo*-N_5_ˉ rings at the equatorial sites. The average Ag1–N distance of 2.519 Å is much longer than the reported values for triazole complexes of Ag(I) (average 2.11 Å)^16^, and the longest bond in the structure, Ag1-N10 (2.669 Å), indicates that the interaction between Ag and *cyclo*-N_5_ˉ is weak.

Energetic metal-organic frameworks (energetic-MOFs) have recently received attention as insensitive HEDMs. The energetic-MOFs are constructed by metal ions and organic ligands, such as azides, furazans, triazoles, and tetrazoles, via coordination bonds, which give one-dimensional (1D), two-dimensional (2D) or three-dimensional (3D) structures^17–20^. Especially noteworthy is the fact that 3D frameworks usually possess more complicated connection modes than 1D and 2D frameworks, which could further enhance their structural stability^21^. As illustrated in Fig. 3c, the silver bridged pentazolate anion in [Ag(NH_3_)_2_]^+^[Ag_3_(N_5_)_4_]ˉ can also be interpreted as a 3D energetic-framework, which is constructed from Ag1, Ag2 and *cyclo*-N_5_ˉ. The overall architecture of [Ag(NH_3_)_2_]^+^[Ag_3_(N_5_)_4_]ˉ is produced with tandem coordination bonding interactions between Ag^+^ and *cyclo*-N_5_ˉ. Continuous catenation in 3D directions is made possible by independent Ag^+^ centers as nodes, coordinatively bound to *cyclo*-N_5_ˉ linkers. The propagation of both six-coordinated Ag1 and four-coordinated Ag2 to *cyclo*-N_5_ˉ generates 3D polycatenated framework (Fig. 3f). Although Ag3 doesnot connect to the 3D framework, the coordinated [Ag(NH_3_)_2_]^+^ is located right in the center of the voids of the crystal structure, and forms hydrogen bonds with the 3D-network (Fig. 3d), (N(11)-H(11 A)···N(2), 2.50 Å; N(11)-H(11 A)···N(8), 2.31 Å; N(11)-H(11B)···N(5), 2.27 Å; N(11)-H(11 C)···N(3), 2.69 Å; N(11)-H(11 C)···N(10), 2.44 Å). To better understand its structure, the 3D framework of [Ag(NH_3_)_2_]^+^[Ag_3_(N_5_)_4_]ˉ can be topologically defined as a 3,4,4,6-c net with long Schlfli symbol of (4•6^2^)_2_(4^2^•6^3^•8)_2_(4^3^•6^3^)_2_(4^4^•6^2^•8^6^•10^3^). As shown in Supplementary Fig. 2, topological analysis indicates that the 3D framework of [Ag(NH_3_)_2_]^+^[Ag_3_(N_5_)_4_]ˉ can be abstracted as a binodal three- and four-connected net, each silver linker connects three or four cyclo-N_5_ˉ anions, which corresponds better to the arrangement of atoms in the 3D framework structure. In addition, typical π–π stacking interactions are observed in [Ag(NH_3_)_2_]^+^[Ag_3_(N_5_)_4_]ˉ between the two off-center parallel *cyclo*-N_5_ˉ rings (Fig. 3e), with centroid–centroid distances of 3.634(5) Å and 3.838(5) Å, respectively, which are consistent with previously reported π–π stacking distances between aromatic molecules^22^. The remarkable face-to-face π–π interactions are important contacts, similar to hydrogen bonding, enhancing the stability of the whole [Ag(NH_3_)_2_]^+^[Ag_3_(N_5_)_4_]ˉ structure. Attempts to determine the surface area and porosity of the 3D framework by Brunner−Emmet−Teller (BET) measurements were unsuccessful because of the inability to completely degas the samples due to their limited thermal stability and the small sample sizes used.

### Physicochemical properties

**Fig. 4:**
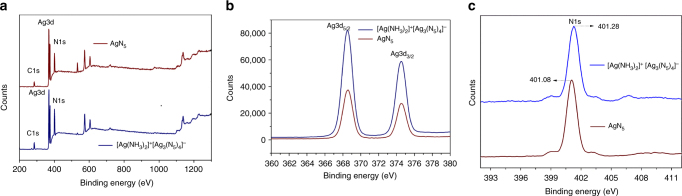
XPS spectra. **a** The wide scan spectra of [Ag(NH_3_)_2_]^+^[Ag_3_(N_5_)_4_]ˉ and the AgN_5_ complex. **b** Core-level Ag3d XPS spectrum. **c** Core-level N1s XPS spectrum

The [Ag(NH_3_)_2_]^+^[Ag_3_(N_5_)_4_]ˉ 3D framework was further investigated by X-ray photoelectron spectroscopy (XPS). Figure 4a shows the XPS wide scan spectrum, which exhibits N1s and Ag3d peaks only. Two peaks at 368.58 and 374.48 eV generated by photoelectrons emitted from the Ag3d core level, can be observed (Fig. 4b), which indicate the presence of only one type of oxidation state for silver that coordinates to the nitrogen atoms in *cyclo*-N_5_ˉ and NH_3_. Figure 4c presents the high-resolution XPS results of N1s. Its binding energy at 401.28 eV is characteristic for the nitrogen atoms that form the *cyclo*-N_5_ˉ ring. These XPS spectra also demonstrate the similarity of the AgN_5_ units in both compounds.

**Fig. 5:**
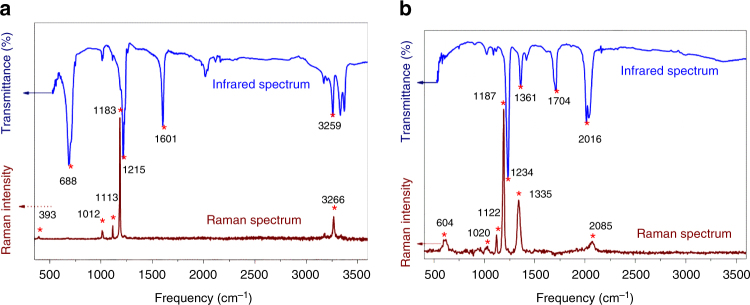
Vibrational spectra. **a** Infrared and Raman spectra of solid [Ag(NH_3_)_2_]^+^[Ag_3_(N_5_)_4_]ˉ. **b** Infrared and Raman spectra of the solid AgN_5_ complex. The red asterisks stand for the position of peak value

We further analyzed the structure of the AgN_5_ complex and [Ag(NH_3_)_2_]^+^[Ag_3_(N_5_)_4_]ˉ by Raman and infrared spectroscopy. As can be seen from Fig. 5, the three typical *cyclo*-N_5_ˉ RA bands are present at about 1180 cm^−1^ (A_1_’), 1120 cm^−1^ (E_2_’) and 1020 cm^−1^ (E_2_’) in both compounds, in excellent agreement with the frequencies observed for (N_5_)_6_(H_3_O)_3_(NH_4_)_4_Cl^1^. For the NH_3_ coordinated cation in [Ag(NH_3_)_2_]^+^[Ag_3_(N_5_)_4_]ˉ (Fig. 5a), two new characteristic bands are observed at 393 and 3266 cm^−1^, which are due to the symmetric Ag-N_2_ stretching mode of [NH_3_-Ag-NH_3_]^+^ and the NH_3_ stretching modes, respectively^23,24^. The infrared spectra of the two compounds show the characteristic absorption of the pentazole rings at ca. 1225 ± 10 cm^−1^ that is generally present in pentazole complexes. The assignments for the NH_3_ bands in [Ag(NH_3_)_2_]^+^[Ag_3_(N_5_)_4_]ˉ are unequivocal, including the different N-H stretching vibrations in the region of 3000–3400 cm^−1^, the symmetric deformation around 1601 cm^−1^, and the rocking mode around 688 cm^−1^. These absorptions of [Ag(NH_3_)_2_]^+^ in [Ag(NH_3_)_2_]^+^[Ag_3_(N_5_)_4_]ˉ agree with those of other diamine silver complexes, such as [Ag(NH_3_)_2_]NO_3_ and [Ag(NH_3_)_2_]_2_SO_4_^25,26^. In the infrared spectrum of the AgN_5_ complex one additional unassigned band is observed at 1704 cm^−1^. In the vibrational spectra of the AgN_5_ complex (Fig. 5b), bands due to N_3_ˉ are observed at 2085, 1335, and 604 cm^−1^ in the RA spectrum (Supplementary Fig. 3), and at 2016 and 1361 cm^−1^ in the infrared (IR) spectrum which are due to N_3_ˉ (ref. ^27^). The fact that the vibrational spectra of the AgN_5_ complex essentially show only bands due to N_5_ˉ and N_3_ˉ lends further support to our identification of this compound as a mixture of solvent-free AgN_5_ and AgN_3_. This conclusion is further supported the crystal structure of [Ag(NH_3_)_2_][Ag_3_(N_5_)_4_], in which no evidence for solvate methanol or water molecules was found. Furthermore, the elemental analysis shows the carbon content in the sample of [Ag(NH_3_)_2_][Ag_3_(N_5_)_4_] to be lower than 0.5%. If some disordered small molecules, such as methanol, existed, they would result in the carbon content to be higher than 0.5%. In addition, no characteristic absorption bands of H_2_O or CH_3_OH were observed in the IR and RA spectra.

The minor slope in the TG curve before 100 °C in the Supplementary Fig. 4 can be attributed to the small sample size and some slight decomposition due to the light-sensitivity of the sample. It is also worth mentioning that there were no endothermic peaks in the differential scanning calorimetry (DSC) curve in the 50~90 °C temperature region, as would be expected for the evaporation of H_2_O or CH_3_OH. The thermal-decomposition behavior and the stability of Ag(NH_3_)_2_]^+^[Ag_3_(N_5_)_4_]ˉ were investigated by thermogravimetric differential scanning calorimetry (TG-DSC) under an argon atmosphere. [Ag(NH_3_)_2_]^+^[Ag_3_(N_5_)_4_]ˉ showed a two-step rapid decomposition beginning at 90 °C with a mass loss of about 25% between 90 and 134 °C, followed by the loss of another approximately 25% between 134 and 320 °C (Supplementary Figs. 4 and 5). Using thermogravimetric analysis, coupled with mass spectroscopy (TG-Mass), a change of the MS curve at mass 17 (NH_3_) was observed along with the release of N_2_ in the first stage of the decomposition (Supplementary Fig. 6). The second step probably involves the decomposition of AgN_3_ to give Ag and N_2_^28^.

**Fig. 6:**
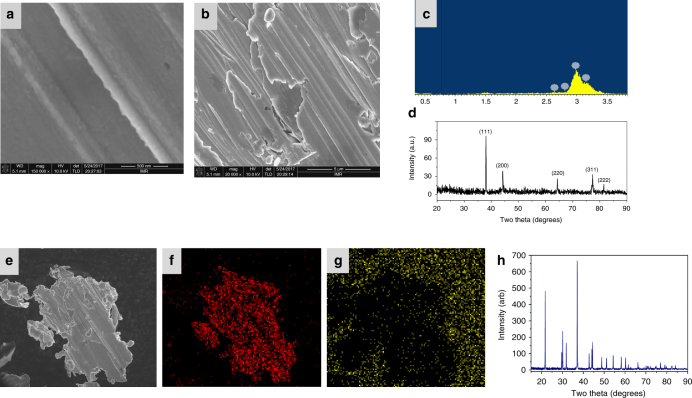
Characterization of the thermal-decomposition residues of [Ag(NH_3_)_2_]^+^[Ag_3_(N_5_)_4_]ˉ. **a** SEM image at low magnification. **b** SEM image at high magnification. **c** EDS spectrum. **d** XRD pattern (JCPDS: 65-2871). **e** SEM image for mapping. **f** EDX mapping distribution of Ag. **g** EDX mapping distribution of N. **h** XRD analysis of the residue from the decomposition process of [Ag(NH_3_)_2_]^+^[Ag_3_(N_5_)_4_]ˉ in the first weight loss step

To confirm the overall decomposition process, the decomposition residue from the first weight loss was investigated by slowly heating the complexes under argon to 100 °C and then cooling them back to room temperature, followed by IR and powder X-ray diffraction (XRD) analyses. The IR spectrum of the [Ag(NH_3_)_2_]^+^[Ag_3_(N_5_)_4_]ˉ residue exhibited the characteristic N_3_ˉ peaks. An additional peak at 3320 cm^−1^ was assigned to HN_3_^29^, suggesting the generation of HN_3_ during the first stage of the decomposition, followed by its absorption on the surface of AgN_3_. In the XRD analysis (Fig. 6h), the position and relative intensity of all diffraction peaks match well with those from a standard AgN_3_ sample, further confirming the composition of the first-step residue as AgN_3._ The XRD powder pattern of the decomposition residue (Fig. 6h) is distinct from that of the original pattern of the starting material before decomposition (Supplementary Fig. 7). One major difference between these complexes and the previously reported (N_5_)_6_(H_3_O)_3_(NH_4_)_4_Cl or Co(N_5_)_2_(H_2_O)_4_·4H_2_O is that during the decomposition silver particles are produced along with complete release of N_2_. The final thermal-decomposition residue from [Ag(NH_3_)_2_]^+^[Ag_3_(N_5_)_4_]ˉ was verified by optical microscopy as pure Ag, which has brilliant metallic luster and an irregular, faceted structure (Supplementary Fig. 8). We have further confirmed this result by using scanning electron microscopy (SEM) and energy dispersive X-ray spectrometry (EDX) to characterize the morphology and determine the chemical phases. Figure 6a indicates that the Ag formed from the thermal-decomposition process consists of multiple nano-layers. Each nano-layer is formed by silver nanoparticles (Fig. 6b), which have small crystallites as evidenced by the XRD analysis. The corresponding intensities of all diffraction peaks are weak due to the relatively low degree of crystallinity (Fig. 6d). The EDX spectrum shows that Ag is the only element detected in the selected region (Fig. 6c), The EDX mappings (Figs. 6e–g) recorded in the whole SEM image indicate that the element on the surface is Ag. By contrast, nitrogen is not observed in the sample region, suggesting the absence of nitrides on the Ag surface. The structure of the AgN_5_ complex was also studied in more detail. The XPS wide scan spectrum of the AgN_5_ complex showed no significant changes compared to that of [Ag(NH_3_)_2_]^+^[Ag_3_(N_5_)_4_]ˉ, indicating a similar chemical composition (except for hydrogen). The core-level spectra of N1s, and Ag3d are presented in the Fig. 4a. The only difference between the AgN_5_ complex and [Ag(NH_3_)_2_]^+^[Ag_3_(N_5_)_4_]ˉ is that the N1s core levels are centered at 401.08 and 401.28 eV, respectively, which illustrates that the presence of different types of nitrogen groups in the AgN_5_ complex has resulted in a slight shift. The IR and RA spectra (Fig. 5b) show only the characteristic peaks of *cyclo*-N_5_ˉ and AgN_3_. To explain the formation of AgN_3_, a sample of the AgN_5_ complex was exposed to light for 24 h, and then the IR spectrum was re-recorded. It was found that the AgN_5_ complex is extremely sensitive to light and completely decomposes to AgN_3_, while the [Ag(NH_3_)_2_]^+^[Ag_3_(N_5_)_4_]ˉ salt is photolytically less sensitive due to the stabilization effect by the 3D framework. In combination with the structure of [Ag(NH_3_)_2_]^+^[Ag_3_(N_5_)_4_]ˉ and the aforementioned data, it, therefore, can be concluded that the AgN_5_ complex is composed of AgN_5_ and AgN_3_. This conclusion was further supported by elemental analysis. The total silver content was determined by inductively coupled plasma optical emission spectroscopy (ICP-OES). The found silver content in the AgN_5_ complex was 62.3 wt%, intermediate between 60.7% (theoretical silver content in AgN_5_) and 72% (theoretical silver content in AgN_3_). The nitrogen content of another sample was also found to be intermediate between the theoretical values for AgN_5_ and AgN_3_. Furthermore, the thermal stability and decomposition behavior of the AgN_5_ complex were also compared to those of [Ag(NH_3_)_2_]^+^[Ag_3_(N_5_)_4_]ˉ. As shown in Supplementary Fig. 9, the TG curve also shows two decomposition stages. The first stage involves loss of N_2_ from AgN_5_ at 120 °C to give AgN_3_, and the second stage comprises the complete decomposition of AgN_3_ at 337 °C to metallic Ag and N_2_.

## Discussion

Our results demonstrate the successful syntheses of a solvent-free silver *cyclo*-pentazolate complex and [Ag(NH_3_)_2_]^+^[Ag_3_(N_5_)_4_]ˉ. The complexes are stable up to 90 °C and only Ag and N_2_ are observed as the final decomposition products. The original product from the [Mg(H_2_O)_6_]^2+^[(N_5_)_2_(H_2_O)_4_]^2^ˉ/AgNO_3_ reaction is AgN_5_, which subsequently undergoes partial photolytical and/or thermal-decomposition to AgN_3_. Although we could not obtain a crystal structure for AgN_5_, the indirect evidence for its formation is convincing. The isolation of a *cyclo*-N_5_ˉ metal complex, devoid of stabilizing molecules and ions, such as H_2_O, H_3_O^+^, and NH_4_^+^, constitutes a major advance in *cyclo*-pentazolate chemistry.

## Methods

### General information

Caution! Solid silver azide and pentazolate are highly energetic and shock and friction sensitive. They should be handled only on a small scale with appropriate safety precautions, i.e., safety glasses, face shields, heavy leather gloves and jackets, and ear plugs.

### Materials characterization

All reagents and solvents used were of analytical grade. (N_5_)_6_(H_3_O)_3_(NH_4_)_4_Cl was produced according to the methods described in the literature^1^. Fourier-transforminfrared spectra were recorded on a Thermo Nicolet IS10 instrument. Raman spectra were measured with a Renishaw (inVia) Raman spectrometer (785 nm excitation). TG-DSC-mass spectrometry (MS) measurements were performed on a Netzsch STA 409 PC/PG thermal analyzer at a heating rate of 5 K/min under argon atmosphere. X-ray photoelectron spectra (XPS) were carried out on a RBD upgraded PHI-5000C electron spectroscopy for chemical analysis (ESCA) system (Perkin Elmer) with Mg *Kα* radiation (hν = 1486.6 eV). The crystalline structure was characterized by X-ray powder diffraction (XRD) with a X-ray diffractometer (D8 advance), using a monochromatized Cu target radiation source. The SEM mapping was observed under SEM (FEI verios 460).

### Synthesis of [Mg(H_2_O)_6_]^2+^[(N_5_)_2_(H_2_O)_4_]^2^ˉ

A solution of Mg(NO_3_)_2_•6H_2_O (0.79 g, 3.08 mmol) in a mixture of solvents (20 mL) of methanol and water (v/v, 1/1) was added to a methanol solution of (N_5_)_6_(H_3_O)_3_(NH_4_)_4_Cl (0.2 g, 0.34 mmol) and stirred at 20 °C for 8 h. The collected filtrate was evaporated under vacuum to furnish a residue. The targeted compound could be recrystallized from the mixture of acetone and methanol and dried in vacuum at room temperature for 4 h to afford the product with an 85% yield of [Mg(H_2_O)_6_]^2+^[(N_5_)_2_(H_2_O)_4_]^2^ˉ as an air-stable white solid.

### Synthesis of [Ag(NH_3_)_2_]^+^[Ag_3_(N_5_)_4_]ˉ

An aqueous solution of silver nitrate (0.34 g, 1.91 mmol) was added dropwise to a solution of [Mg(H_2_O)_6_]^2+^[(N_5_)_2_(H_2_O)_4_]^2^ˉ (0.3 g, 0.87 mmol) in methanol while stirring at 20 °C for 30 min, producing the silver pentazolate complex as a pale solid. It was quickly dissolved in 10 equiv. of NH_4_OH and stirred at 0 °C for 20 min, followed by warming to room temperature to liberate NH_3_, providing the target product, [Ag(NH_3_)_2_]^+^[Ag_3_(N_5_)_4_]ˉ, in 80% yield as an air-stable white solid.

### Data availability

The authors declare that the data supporting the findings of this study are available within the article and its Supplementary Information files. All other relevant data supporting the findings of this study are available on request. Structural data for [Ag(NH_3_)_2_]^+^[Ag_3_(N_5_)_4_]ˉ and [Mg(H_2_O)_6_]^2+^[(N_5_)_2_(H_2_O)_4_]^2^ˉ were deposited with the Inorganic Crystal Structure Database (ICSD) under deposition numbers CSD: 433114 and 433851, respectively.

## Electronic supplementary material

Supplementary Information(PDF 504 kb)
