# Supplementary material for: Synthesis of AgN5 and its extended 3D energetic framework
Source: Nat Commun. 2018 Mar 28;9:1269. doi: 10.1038/s41467-018-03678-y (PMC5871778; doi:10.1038/s41467-018-03678-y)
Supplement: Supplementary file 1 — Supplementary Information(PDF 504 kb) [file 41467_2018_3678_MOESM1_ESM.pdf]

Synthesis of AgN_5 and its extended 3D energetic framework

Chengguo Sun et al.

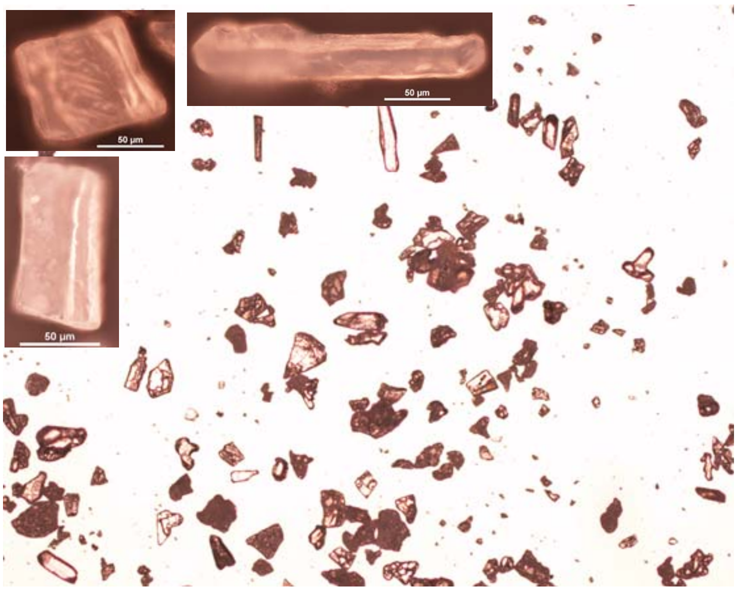

Supplementary Figure 1 | Optical photographs of $[Ag(NH_3)_2]^+[Ag_3(N_5)_4]^-$ crystals. Three crystals are enlarged for illustration**.**

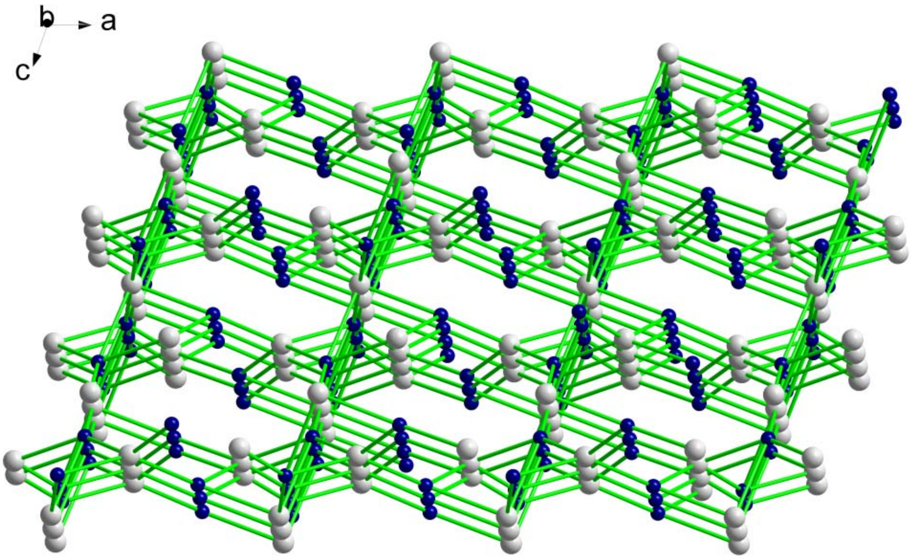

Supplementary Figure 2 | Topological representation of the 3D framework in $[Ag(NH_3)_2]^+[Ag_3(N_5)_4]^-$.

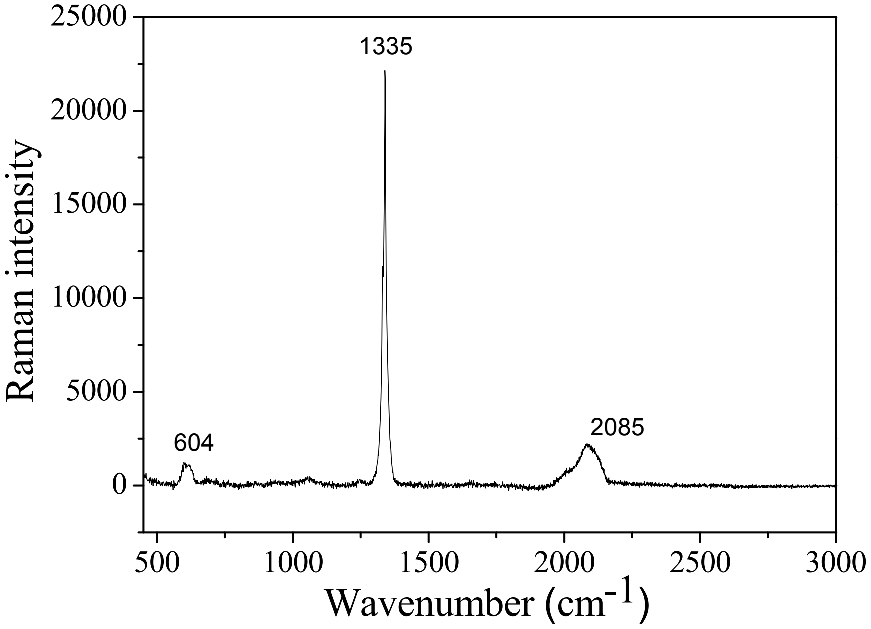

Supplementary Figure 3 | Raman spectrum of an AgN_3 reference sample.

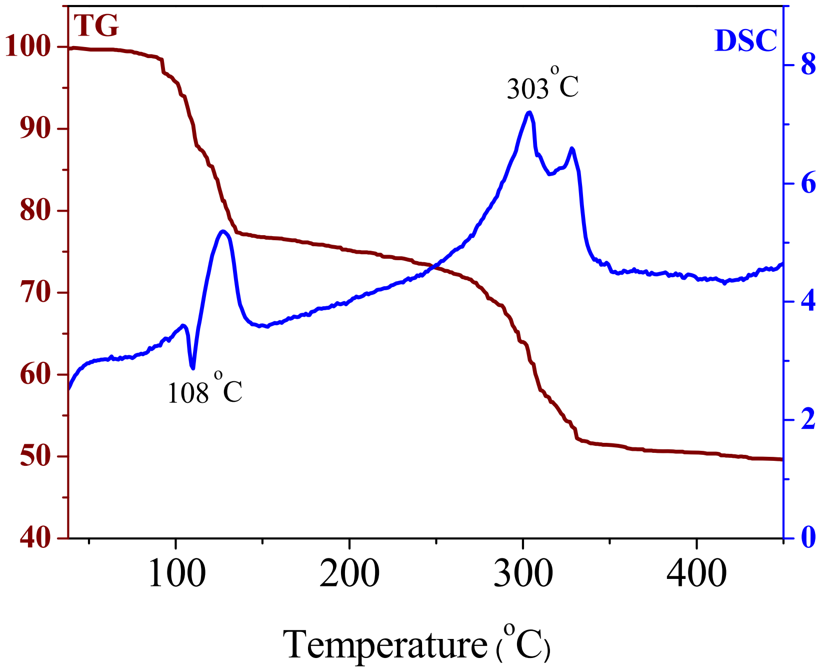

Supplementary Figure 4 | TG-DSC curves of $[Ag(NH_3)_2]^+[Ag_3(N_5)_4]^-$ under an argon atmosphere at 5 K/min.

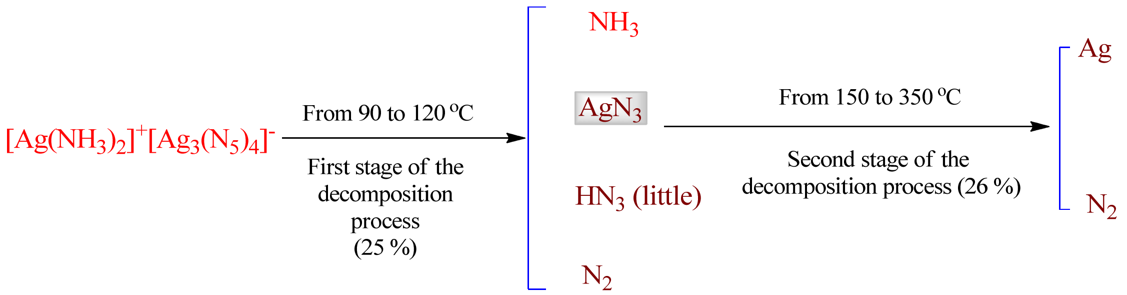

Supplementary Figure 5 | The proposed thermal decomposition process of $[Ag(NH_3)_2]^+[Ag_3(N_5)_4]^-$.

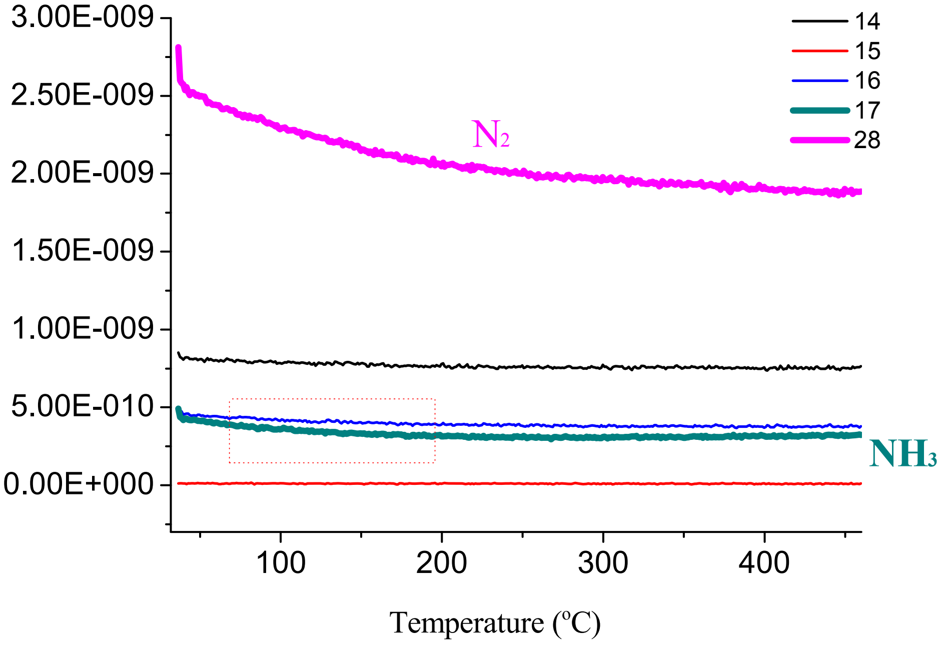

Supplementary Figure 6 | The mass spectra of selected ion monitoring scan of the gas products obtained from the thermal decomposition of $[Ag(NH_3)_2]^+[Ag_3(N_5)_4]^-$ under argon.

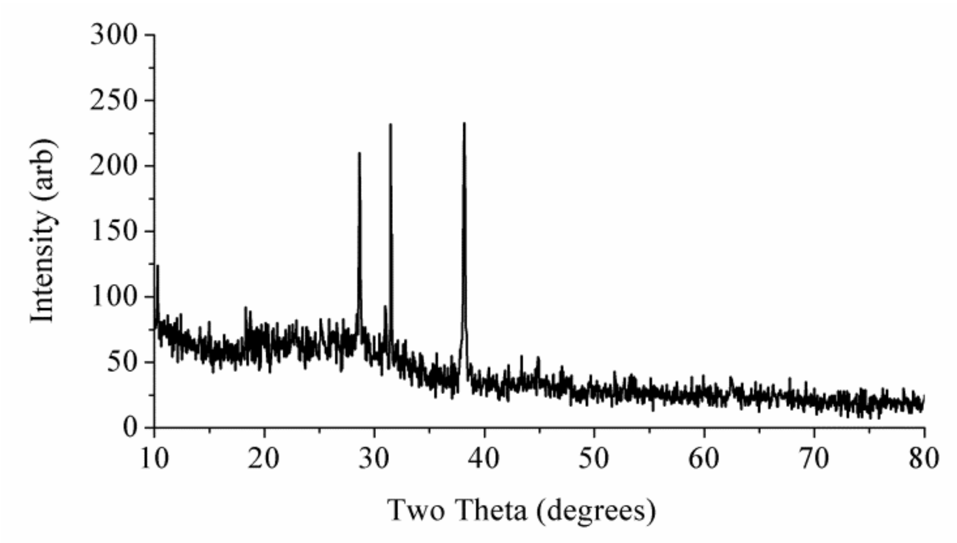

Supplementary Figure 7 | The XRD powder pattern of $[Ag(NH_3)_2]^+[Ag_3(N_5)_4]^-$ before thermal decomposition.

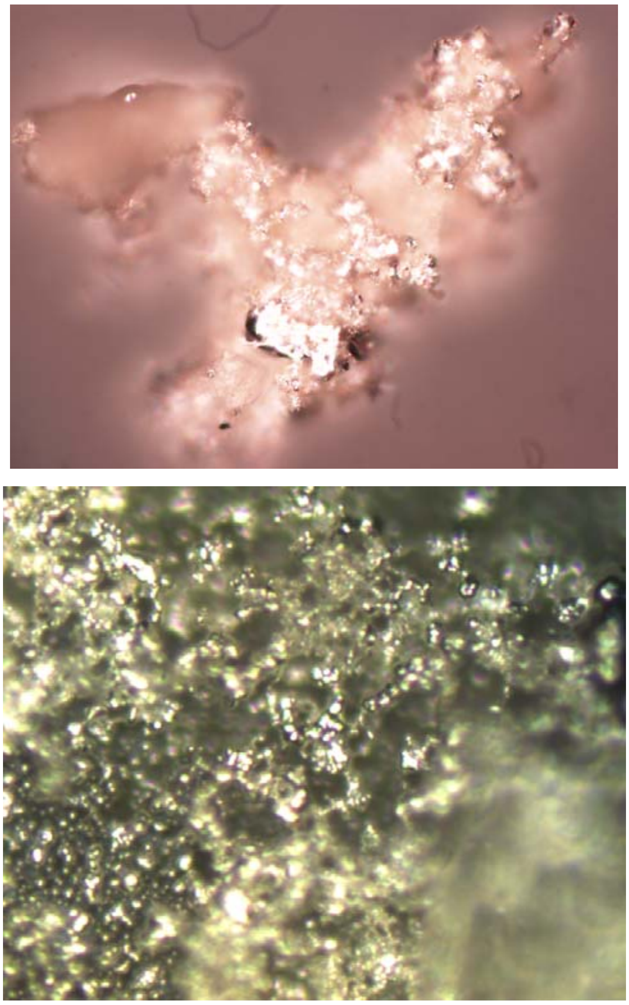

Supplementary Figure 8 | Optical photographs of the thermal-decomposition residues of $[Ag(NH_3)_2]^+[Ag_3(N_5)_4]^-$.

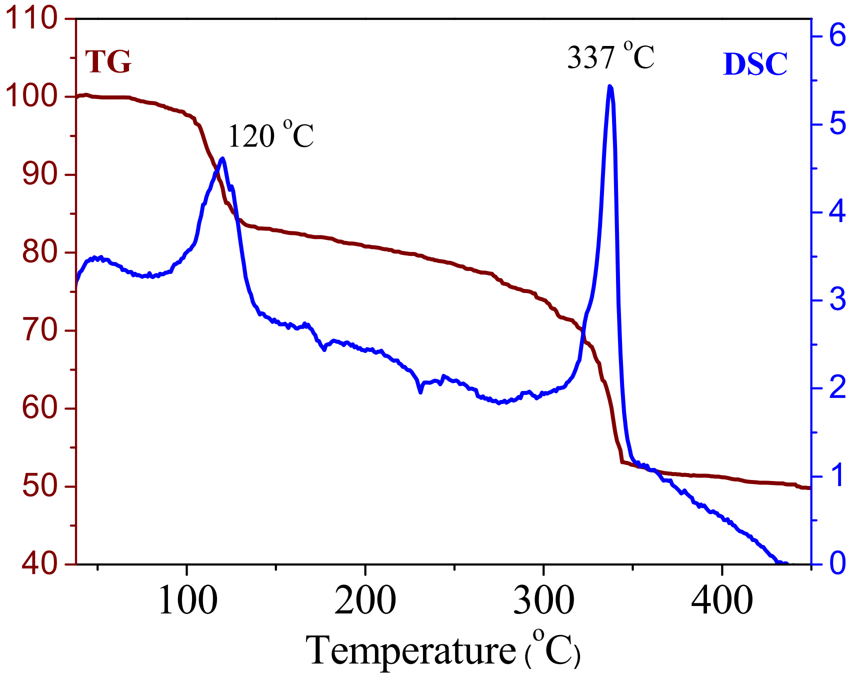

Supplementary Figure 9 | TG-DSC curves of the AgN_5 complex under an argon atmosphere at 5 K/min.

Supplementary Table 1 | The impact sensitivity Friction sensitivity of $[Ag(NH_3)_2]^+[Ag_3(N_5)_4]^-$ and comparison with Nano-CL-20, HMX and RDX

Sample	$[Ag(NH_3)_2]^+[Ag_3(N_5)_4]^-$	Nano CL-20[c]	RDX (5-class)[d]	spherical β-HMX[e]
H_{50} value (cm)	73.8[a]	29.4	21.8	54.1
Explosive probability P (%)=76	76[b]	66	80	80

[a]: Testing Conditions.Test temperature:24 °C, Relative humidity: 60%, Hammer weight: 2.0 kg.

[b] Testing Conditions. Friction instrument: BM-B 20Z126, Test temperature: 24 °C, Relative humidity: 60%, Sample weight :20mg, Pressure:2.45 Mpa; Angle: 66°.

[c] Supplementary Reference 1

[d] Supplementary Reference 2

[e] Supplementary Reference 3

Supplementary Table 2 | Crystallographic data for $[Ag(NH_3)_2]^+[Ag_3(N_5)_4]^-$

	$Ag_4H_6N_{22}$
Formula	$[Ag(NH_3)_2]^+[Ag_3(N_5)_4]^-$
Formula weight	745.75 g·mol^{-1}
Temperature	123.15 K
Wavelength	0.71073 Å
Crystal system	Monoclinic
Space group	$P2_1/c$
Cell parameters	a= 9.1248(18) Å b= 9.1238(18) Å c=9.8307(19) Å
	α=90° β=108.963(5)° γ=90°
Cell volume	774.0(3) Å^3
Formula Z	2
Calc. density	3.200 g·cm^{-3}
Absorption coefficient	5.032 mm^{-1}
F(000)	696
Crystal size	0.22 × 0.25 × 0.26 mm
Theta range for data collection	2.360 to 26.366°
Limiting indices	-11<=h<=11, -11<=k<=10, -12<=l<=12
Reflections collected / unique	5590/1554 [R(int) = 0.0468]
Absorption correction	Semi-empirical from equivalents
Max. and min. transmission	0.7457 and 0.5708
Refinement method	Full-matrix least-squares on F^2
Goodness-of-fit on F^2	1.142
Final R indices [I>2sigma(I)]	R_1 = 0.0310, wR_2 = 0.0799
R indices (all data)	R_1 = 0.0333, wR_2 = 0.0817
Largest diff. peak and hole	0.648 and -1.243 e·Å^{-3}
CSD	433114

Supplementary Table 3 | Bond lengths.

parameter	bond length(Å)	parameter	bond length(Å)
Ag(1)-N(3)	2.375(3)	N(2)-N(3)	1.323(4)
Ag(1)-N(3)$^{\#1}$	2.375(3)	N(3)-N(4)	1.330(4)
Ag(1)-N(7)$^{\#2}$	2.513(3)	N(4)-Ag(2)$^{\#4}$	2.370(3)
Ag(1)-N(7)$^{\#3}$	2.513(3)	N(4)-N(5)	1.323(4)
Ag(1)-N(10)$^{\#4}$	2.669(3)	N(6)-N(7)	1.327(4)
Ag(1)-N(10)$^{\#5}$	2.669(3)	N(6)-N(10)	1.330(4)
Ag(3)-N(11)	2.110(3)	N(7)-Ag(1)$^{\#9}$	2.513(3)
Ag(3)-N(11)$^{\#6}$	2.110(3)	N(7)-N(8)	1.320(4)
Ag(2)-N(1)	2.332(3)	N(8)-N(9)	1.318(4)
Ag(2)-N(4)$^{\#7}$	2.370(3)	N(9)-Ag(2)$^{\#10}$	2.365(3)
Ag(2)-N(6)	2.336(3)	N(9)-N(10)	1.328(4)
Ag(2)-N(9)$^{\#8}$	2.365(3)	N(11)-H(11A)	0.9100
N(1)-N(2)	1.327(4)	N(11)-H(11B)	0.9100
N(1)-N(5)	1.336(4)	N(11)-H(11C)	0.9100

Symmetry code: #1: -x+2, -y+1, -z+1 #2: -x+1, -y+1, -z+1 #3: x+1, y, z #4: -x+1, y-1/2, -z+1/2 #5: x+1, -y+3/2, z+1/2 #6: -x+1, -y+2, -z+1 #7: -x+1, y+1/2, -z+1/2 #8: -x, y-1/2, -z+1/2 #9: x-1, y, z #10: -x, y+1/2, -z+1/2.

Supplementary Table 4 | Bond angles.

parameter	bond angle(°)	parameter	bond angle(°)
N(3)-Ag(1)-N(3)$^{\#1}$	180.0	N(3)-N(2)-N(1)	107.2(3)
N(3)$^{\#1}$-Ag(1)-N(7)$^{\#3}$	85.55(9)	N(2)-N(3)-Ag(1)	125.8(2)
N(3)-Ag(1)-N(7)$^{\#3}$	94.45(9)	N(2)-N(3)-N(4)	108.4(2)
N(3)$^{\#1}$-Ag(1)-N(7)$^{\#2}$	94.45(9)	N(4)-N(3)-Ag(1)	125.3(2)
N(3)-Ag(1)-N(7)$^{\#2}$	85.55(9)	N(3)-N(4)-Ag(2)$^{\#4}$	124.62(19)
N(3)-Ag(1)-N(10)$^{\#4}$	86.51(9)	N(5)-N(4)-Ag(2)$^{\#4}$	121.1(2)
N(3)$^{\#1}$-Ag(1)-N(10)$^{\#5}$	86.50(9)	N(5)-N(4)-N(3)	108.6(3)
N(3)$^{\#1}$-Ag(1)-N(10)$^{\#4}$	93.50(9)	N(4)-N(5)-N(1)	106.8(3)
N(3)-Ag(1)-N(10)$^{\#5}$	93.49(9)	N(7)-N(6)-Ag(2)	128.6(2)
N(7)$^{\#2}$-Ag(1)-N(7)$^{\#3}$	180.0	N(7)-N(6)-N(10)	107.7(2)
N(7)$^{\#2}$-Ag(1)-N(10)$^{\#5}$	85.18(9)	N(10)-N(6)-Ag(2)	122.8(2)
N(7)$^{\#3}$-Ag(1)-N(10)$^{\#5}$	94.82(9)	N(6)-N(7)-Ag(1)$^{\#9}$	123.4(2)
N(7)$^{\#2}$-Ag(1)-N(10)$^{\#4}$	94.82(9)	N(8)-N(7)-Ag(1)$^{\#9}$	123.8(2)
N(7)$^{\#3}$-Ag(1)-N(10)$^{\#4}$	85.18(9)	N(8)-N(7)-N(6)	108.7(3)
N(10)$^{\#4}$-Ag(1)-N(10)$^{\#5}$	180.0	N(9)-N(8)-N(7)	107.4(2)
N(11)-Ag(3)-N(11)$^{\#6}$	180.00(13)	N(8)-N(9)-Ag(2)$^{\#10}$	128.4(2)
N(1)-Ag(2)-N(4)$^{\#7}$	97.19(9)	N(8)-N(9)-N(10)	109.0(3)
N(1)-Ag(2)-N(6)	141.14(9)	N(10)-N(9)-Ag(2)$^{\#10}$	122.6(2)
N(1)-Ag(2)-N(9)$^{\#8}$	93.97(9)	N(9)-N(10)-N(6)	107.2(3)
N(6)-Ag(2)-N(4)$^{\#7}$	99.67(9)	Ag(3)-N(11)-H(11A)	109.5
N(6)-Ag(2)-N(9)$^{\#8}$	95.60(10)	Ag(3)-N(11)-H(11B)	109.5
N(9)$^{\#8}$-Ag(2)-N(4)$^{\#7}$	139.30(9)	Ag(3)-N(11)-H(11C)	109.5
N(2)-N(1)-Ag(2)	128.2(2)	H(11A)-N(11)-H(11B)	109.5
N(2)-N(1)-N(5)	109.0(2)	H(11A)-N(11)-H(11C)	109.5
N(5)-N(1)-Ag(2)	121.9(2)	H(11B)-N(11)-H(11C)	109.5

Symmetry code: #1: -x+2, -y+1, -z+1 #2: -x+1, -y+1, -z+1 #3: x+1, y, z #4: -x+1, y-1/2, -z+1/2 #5: x+1, -y+3/2, z+1/2 #6: -x+1, -y+2, -z+1 #7: -x+1, y+1/2, -z+1/2 #8: -x, y-1/2, -z+1/2 #9: x-1, y, z #10: -x, y+1/2, -z+1/2.

Supplementary Table 5 | Hydrogen bonds for $[Ag(NH_3)_2]^+[Ag_3(N_5)_4]^-$.

D-H...A	D-H(Å)	H…A(Å)	D…A(Å)	D-H…A(°)
N(11)-H(11A)...N(2)	0.91	2.50	3.079(4)	122.1
N(11)-H(11A)...N(8)$^{\#3}$	0.91	2.31	3.112(4)	146.6
N(11)-H(11B)...N(5)$^{\#2}$	0.91	2.27	3.152(4)	162.1
N(11)-H(11C)...N(3)$^{\#11}$	0.91	2.69	3.289(4)	124.2
N(11)-H(11C)...N(10)$^{\#6}$	0.91	2.44	3.257(4)	148.7

Symmetry code: #1: -x+2, -y+1, -z+1 #2: -x+1, -y+1, -z+1 #3: x+1, y, z #4: -x+1, y-1/2, -z+1/2 #5: x+1, -y+3/2, z+1/2 #6: -x+1, -y+2, -z+1 #7: -x+1, y+1/2, -z+1/2 #8: -x, y-1/2, -z+1/2 #9: x-1, y, z #10: -x, y+1/2, -z+1/2 #11: x, -y+3/2, z+1/2.

Supplementary Table 6 | Torsion angles.

parameter	torsion angle(°)	parameter	torsion angle(°)
Ag(1)-N(3)-N(4)-Ag(2)$^{\#4}$	-18.9(3)	N(2)-N(1)-N(5)-N(4)	0.0(3)
Ag(1)-N(3)-N(4)-N(5)	-172.3(2)	N(2)-N(3)-N(4)-Ag(2)$^{\#4}$	153.5(2)
Ag(1)$^{\#9}$-N(7)-N(8)-N(9)	-158.2(2)	N(2)-N(3)-N(4)-N(5)	0.0(3)
Ag(2)-N(1)-N(2)-N(3)	-168.9(2)	N(3)-N(4)-N(5)-N(1)	0.0(3)
Ag(2)-N(1)-N(5)-N(4)	169.74(18)	N(5)-N(1)-N(2)-N(3)	0.0(3)
Ag(2)$^{\#4}$-N(4)-N(5)-N(1)	-154.6(2)	N(6)-N(7)-N(8)-N(9)	-0.3(4)
Ag(2)-N(6)-N(7)-Ag(1)$^{\#9}$	-10.5(4)	N(7)-N(6)-N(10)-N(9)	-0.4(4)
Ag(2)-N(6)-N(7)-N(8)	-168.5(2)	N(7)-N(8)-N(9)-Ag(2)$^{\#10}$	-179.4(2)
Ag(2)-N(6)-N(10)-N(9)	169.3(2)	N(7)-N(8)-N(9)-N(10)	0.1(4)
Ag(2)$^{\#10}$-N(9)-N(10)-N(6)	179.7(2)	N(8)-N(9)-N(10)-N(6)	0.2(4)
N(1)-N(2)-N(3)-Ag(1)	172.25(19)	N(10)-N(6)-N(7)-Ag(1)$^{\#9}$	158.5(2)
N(1)-N(2)-N(3)-N(4)	0.0(3)	N(10)-N(6)-N(7)-N(8)	0.4(4)

Symmetry code: #1: -x+2, -y+1, -z+1 #2: -x+1, -y+1, -z+1 #3: x+1, y, z #4: -x+1, y-1/2, -z+1/2 #5: x+1, -y+3/2, z+1/2 #6: -x+1, -y+2, -z+1 #7: -x+1, y+1/2, -z+1/2 #8: -x, y-1/2, -z+1/2 #9: x-1, y, z #10: -x, y+1/2, -z+1/2.

Supplementary References

1. Guo, X., Ouyang, G., Liu, J., Li, Q., Wang, L., Gu, Z., & Li, F. Massive preparation of reduced-sensitivity nano CL-20 and its characterization. *J. Energ. Mater.*, **33(1)**, 24-33 (2015).

2. Jiang, X., Guo, X., Ren, H., & Jiao, Q. Preparation and characterization of desensitized ε-HNIW in solvent-antisolvent recrystallizations. *Cent. Eur. J. Energ. Mater.,* **9(3)**, 219-236 (2012).

3. Song, X., Wang, Y., An, C., Guo, X., & Li, F.. Dependence of particle morphology and size on the mechanical sensitivity and thermal stability of octahydro-1, 3, 5, 7-tetranitro-1, 3, 5, 7-tetrazocinCompeting interests: The authors declare no competing financial interests.e. *J. Hazard. Mater.*, **159(2-3)**, 222-229 (2008).
